# The German Quality Network Sepsis: Evaluation of a Quality Collaborative on Decreasing Sepsis-Related Mortality in a Controlled Interrupted Time Series Analysis

**DOI:** 10.3389/fmed.2022.882340

**Published:** 2022-04-27

**Authors:** Daniel Schwarzkopf, Hendrik Rüddel, Alexander Brinkmann, Carolin Fleischmann-Struzek, Marcus E. Friedrich, Michael Glas, Christian Gogoll, Matthias Gründling, Patrick Meybohm, Mathias W. Pletz, Torsten Schreiber, Daniel O. Thomas-Rüddel, Konrad Reinhart

**Affiliations:** ^1^Integrated Research and Treatment Center for Sepsis Control and Care (CSCC), Jena University Hospital, Jena, Germany; ^2^Department for Anesthesiology and Intensive Care Medicine, Jena University Hospital, Jena, Germany; ^3^Institute for Infectious Diseases and Infection Control, Jena University Hospital, Jena, Germany; ^4^Department of Anesthesiology and Intensive Care Medicine, General Hospital of Heidenheim, Heidenheim, Germany; ^5^New York State Department of Health, Albany, NY, United States; ^6^Department for Infectious Diseases and Infection Control, KH Labor GmbH, AMEOS Group, Bernburg, Germany; ^7^Outpatient Services, Evangelische Lungenklinik Berlin-Buch, Berlin, Germany; ^8^Department of Anesthesiology, University Hospital of Greifswald, Greifswald, Germany; ^9^Department of Anesthesiology, Intensive Care, Emergency and Pain Medicine, University Hospital Wuerzburg, Wuerzburg, Germany; ^10^Department of Anesthesia and Intensive Care, Zentralklinik Bad Berka, Bad Berka, Germany; ^11^Berlin Institute of Health, Campus Virchow-Klinikum, Berlin, Germany; ^12^Department of Anesthesiology and Operative Intensive Care Medicine (CCM, CVK), Charité – Universitätsmedizin Berlin, Corporate Member of Freie Universität Berlin, Humboldt-Universität zu Berlin, Berlin, Germany

**Keywords:** sepsis, mortality, quality improvement, risk adjustment, administrative claims, interdisciplinary health team, diagnosis-related groups (DRG)

## Abstract

**Background:**

Sepsis is one of the leading causes of preventable deaths in hospitals. This study presents the evaluation of a quality collaborative, which aimed to decrease sepsis-related hospital mortality.

**Methods:**

The German Quality Network Sepsis (GQNS) offers quality reporting based on claims data, peer reviews, and support for establishing continuous quality management and staff education. This study evaluates the effects of participating in the GQNS during the intervention period (April 2016–June 2018) in comparison to a retrospective baseline (January 2014–March 2016). The primary outcome was all-cause risk-adjusted hospital mortality among cases with sepsis. Sepsis was identified by International Classification of Diseases (ICD) codes in claims data. A controlled time series analysis was conducted to analyze changes from the baseline to the intervention period comparing GQNS hospitals with the population of all German hospitals assessed *via* the national diagnosis-related groups (DRGs)-statistics. Tests were conducted using piecewise hierarchical models. Implementation processes and barriers were assessed by surveys of local leaders of quality improvement teams.

**Results:**

Seventy-four hospitals participated, of which 17 were university hospitals and 18 were tertiary care facilities. Observed mortality was 43.5% during baseline period and 42.7% during intervention period. Interrupted time-series analyses did not show effects on course or level of risk-adjusted mortality of cases with sepsis compared to the national DRG-statistics after the beginning of the intervention period (*p* = 0.632 and *p* = 0.512, respectively). There was no significant mortality decrease in the subgroups of patients with septic shock or ventilation >24 h or predefined subgroups of hospitals. A standardized survey among 49 local quality improvement leaders in autumn of 2018 revealed that most hospitals did not succeed in implementing a continuous quality management program or relevant measures to improve early recognition and treatment of sepsis. Barriers perceived most commonly were lack of time (77.6%), staff shortage (59.2%), and lack of participation of relevant departments (38.8%).

**Conclusion:**

As long as hospital-wide sepsis quality improvement efforts will not become a high priority for the hospital leadership by assuring adequate resources and involvement of all pertinent stakeholders, voluntary initiatives to improve the quality of sepsis care will remain prone to failure.

## Introduction

Sepsis is a life-threatening organ dysfunction resulting from infection and the leading cause of death due to infectious diseases (1). It might also be the leading cause of preventable deaths in hospitals (2). Timely recognition and adequate anti-infective treatment have been shown to decrease mortality, but awareness of sepsis is often low in everyday clinical practice (2–8). A recent meta-analysis showed that performance improvement programs substantially improved implementation of sepsis guidelines including early adequate antimicrobial treatment – and decreased odds of death (9). Such quality initiatives typically use a multifaceted approach by assessing and reporting quality, staff education, and implementing changes in care processes (9). Prospective inclusion of patients with sepsis and documentation of clinical data for quality indicators put a high workload on participating hospitals, which can cause poor reporting or even the drop-out of hospitals from quality improvement projects (10, 11).

Using claims data for performance measurement has the advantage of covering all International Classification of Diseases (ICD) coded cases with data readily available and needing minimal time and costs (12). This approach is extensively used within quality initiatives in the United States of America (USA) (13). It has also achieved the first promising results in Germany, where a large quality initiative combines benchmarks of quality indicators based on administrative data with peer reviews (14). Therefore, the German Quality Network Sepsis (GQNS) was founded as a quality collaborative to support participating hospitals to improve sepsis care by offering quality reports based on claims data, peer reviews, and support to implement a continuous quality management and regular staff education. The participation in the GQNS was voluntary and the full responsibility for implementation of quality improvement measures was on the side of the participating hospitals. This study aims to evaluate the effect of hospitals’ participation in the GQNS on mortality among patients with sepsis.

## Materials and Methods

### Context

The GQNS was founded in February 2016. The start-up period of the GQNS was funded by grants from the German Federal Ministry of Education and Research (BMBF) and ran from August 2015 to July 2018. The funded start-up phase and its scientific evaluation used the acronym quality Improvement in infection COntrol and Sepsis management in MOdel regionS (ICOSMOS). The study was approved by the Ethical Review Board of the Jena University Hospital (IRB protocol 4536-08/15). The necessity of informed consent by patients was waived since only pseudonymized claims data were used. Details on the concept and conduction of the GQNS, as well as the planned evaluation, are given in the study protocol (15). Passages cited from the study protocol are not individually marked in the manuscript. The study description follows the Standards for QUality Improvement Reporting Excellence (SQUIRE 2.0) recommendations (16).

### Participating Hospitals

Eligible for participation in the GQNS were acute care hospitals with at least one adult intensive care unit. Invitation letters were sent to management boards of hospitals that were participating in former or ongoing sepsis-related quality initiatives or research networks and all German university hospitals; a total of 148 individual hospitals were contacted. In addition, letters were sent to management boards of five regional and three national hospital groups. Hospitals could join the GQNS at the time of its foundation or any later time.

### Project Organization

The GQNS was coordinated by the central study coordinating bureau at the Jena University Hospital. Claims data were collected and processed to generate quality reports by a medical information technology service provider (3M Health Information Systems). Participating hospitals of the GQNS named a local leader of the quality improvement process. The quality improvement leaders were encouraged to establish interdisciplinary and interprofessional quality improvement teams right from the beginning of the participation in the GQNS. The formation of these teams was not mandatory and selection of the members was at the discretion of the quality improvement leader. It was suggested by the study coordinating bureau to include at least intensive care departments, the emergency department, quality management department, and medical and surgical departments responsible for inpatient treatments of adult patients. Major decisions were made in the general assembly of representatives of all participating hospitals. This general assembly was formed by the local quality improvement leaders and met once a year in autumn. A steering committee was elected among the delegates of the general assembly to supervise the work of the coordinating bureau. Meetings of the steering committee and the study coordinating bureau were conducted by phone or web-conference every few months.

### Interventions

The core interventions for hospitals in the GQNS are: (a) reporting and publication of quality indicators; (b) case analyses within the participating hospitals; (c) peer reviews for hospitals, which were outliers in the quality reports; and (d) hospital-wide staff education in participating hospitals. Peer review is a process by which health care providers evaluate each other’s performance (17). The only mandatory intervention was the reporting, benchmarking, and publication of quality indicators. The study coordination bureau provided information and support regarding the conduction of case analyses and staff education and coordinated peer reviews. The full responsibility for implementation was on the side of the participating hospitals, and the participation in peer reviews was voluntary.

#### Reporting of Quality Indicators

Data for assessment of quality indicators were provided by diagnosis-related group (DRG) data of each participating hospital, which were sent to the information technology service provider. These data can be exported easily in a standardized format from the hospitals’ patient data management system. The service provider supplied the quality reporting quarterly to each hospital beginning in April 2016. Cases with sepsis were identified based on specific codes of the International Statistical Classification of Diseases and Related Health Problems 10th Revision German Modification (ICD-10-GM) for sepsis with organ dysfunction or septic shock according to sepsis-1 definitions (R65.1: sepsis with organ dysfunction, R57.2: septic shock) (18). Although new clinical sepsis definitions (“sepsis-3”) were introduced in 2016 (1), the ICD-10-GM-coding of sepsis relied on the old sepsis-1 definitions until the end of 2019 in Germany. Quality reports contained incidence and risk-adjusted mortality for cases with sepsis and the subgroups of patients with septic shock, sepsis, and mechanical ventilation of more than 24 h, admission to the hospital *via* a surgical department, and admission to the hospital *via* a medical department. Hospitals’ own results could be compared to other participating hospitals, subgroups of participating hospitals (primary, secondary, tertiary care, and university hospitals), the overall average in the GQNS, as well as to the average among all German hospitals. Also the longitudinal course of quality indicators could be inspected using monthly, quarterly, half-yearly, and yearly periods. Initially, quality reports were presented in tabular form by Microsoft Excel spreadsheets. From July 2017 onward, quality indicators were additionally reported in an online-reporting accessed *via* a web-browser. This online-reporting also contained interactive graphical presentations of quality indicators, e.g., boxplots, and caterpillar plots. Both calculation and presentation of quality indicators were continuously improved.

Mortality was risk-adjusted by a validated complex model developed for the GQNS, which was based on German national DRG-statistics (19). This database contains DRG-data of all German hospitals that are reimbursed *via* DRG. It is provided for scientific analysis in anonymized form by the German Federal Bureau of Statistics (20). Therefore, the same type of data, which are provided by the GQNS-hospitals for quality measurement, are available in the national DRG-statistics and patients with sepsis were identified by the same criteria as given above. The detailed development and validation of the risk-model is described elsewhere (19). Included risk-factors are age, gender, type of admission, clinical characteristics of infection and sepsis, comorbidities, and specific procedures – like treatment of stroke (19). Definitions of variables for risk-adjustment and quality reporting are presented in Supplementary Data Sheet 1.

Quality reporting also included case lists presenting predicted and observed mortality for each sepsis case sent to the hospitals, which provided the basis for case analysis and peer reviews. The study coordinating bureau provided hospitals with instructions on how to use the quality reports, and how to conduct case analyses. This was done during annual meetings and by providing educational material on the website of the GQNS.

#### Publication of Quality Indicators

Hospitals within the GQNS consented to publish their major quality indicators compared to the average of the German national DRG-statistics on their own website. Two indicators were to be published: risk-adjusted mortality of patients with sepsis, and risk-adjusted mortality of patients with sepsis and mechanical ventilation >24 h. To allow hospitals to analyze their data as well as to learn and implement improvements, the first publication of quality indicators was mandatory after 2 years of participation in the GQNS. Therefore, there was one publication of quality indicators at the end of the start-up period of the GQNS in Summer of 2018. All hospitals, which had signed their contract for participation in the GQNS in 2015 were obliged to publish their quality indicators of the year 2017. This was the case for 11 hospitals.

#### Case Analyses and Peer Reviews

Based on the provided case lists, expired patients with sepsis with the lowest risk of in-hospital mortality as predicted by the risk-adjustment model were identified and used to analyze and discuss possible problems in the quality of care in interdisciplinary case conferences within the individual participating hospitals (21). The same method was used to select cases for analysis by external peers. An external peer review was suggested by the central study coordinating bureau to hospitals with the highest risk-adjusted mortality among patients with sepsis. Peers were physicians and nurses, who were recruited among the participating hospitals and had a special qualification to conduct peer reviews. A team of at least four peers visited the respective hospital, conducted analyses of up to 10 selected charts of patients with sepsis, and discussed improvement strategies with local clinicians. Contents and results of peer reviews were only reported to the participating hospital and the central study coordinating bureau. Peer reviews were voluntary and hospitals could refuse to take part. Six peer-reviews were conducted from May 2017 to April 2018.

#### Staff Education

The main focus of staff education was the implementation of strategies for increasing awareness and early recognition of sepsis, as well as the implementation of key elements of the updated Surviving Sepsis Campaign guidelines among all health care workers involved in care for patients with sepsis (22, 23). The study coordination bureau supported the local hospital quality improvement leaders by providing educative material (presentations, pocket cards, posters). Hospitals were also provided with a concept for a screening algorithm for the early detection of sepsis as well as recommendations for its implementation. Educational materials were provided for download *via* the website of the GQNS and concepts were presented at the annual meetings. The local quality improvement teams were responsible for implementing education. In addition, five web-based educational sessions were conducted between March 2017 and February 2018, recordings of these sessions were provided on the website of the GQNS. Due to overall low participation rates and technical problems reported by many participants, no further web based sessions were done. Further details on the interventions are provided in the study protocol (15).

### Evaluation of the Effect of Participating in the German Quality Network Sepsis

The effect of participating in the GQNS was evaluated in a controlled interrupted time series analysis (24). The start of the intervention was defined individually for each participating hospital as the month of supply of the first quality report. Thus, for each hospital an individual baseline period and an individual intervention period was defined. This allowed to use all available information of all participating hospitals. The retrospective baseline period began in January 2014 and ended when the hospital received its first quality report. Most hospitals switched to intervention in April 2016; the analyzed intervention period ended, when a hospital stopped its participation in the GQNS or with June 2018 – the time point of the latest delivered DRG-data. Since this analysis might be biased by seasonal variation or history bias, a control condition was included (24). As the control condition, the German national DRG-statistics was used to calculate the monthly risk-standardized mortality rate (RSMR) for all coded sepsis cases in Germany (20), which can be regarded as the population value.

#### Outcome Measures

The evaluation was based on the data of the quality reports, which were provided to the research team by the medical information technology service provider. Due to data privacy restrictions, no data of individual cases were provided, but all data were aggregated to the hospital level. The primary outcome was the monthly risk-adjusted hospital mortality per hospital of cases with primary or secondary hospital discharge ICD-10-GM code for sepsis with organ dysfunction including septic shock (R65.1, R57.2). Secondary outcomes were the risk-adjusted mortality among patients with septic shock (ICD-10-GM code R57.2) and among cases with sepsis and mechanical ventilation of more than 24 h. Risk-adjusted mortality was calculated as RSMR (see Supplementary Data Sheet 1 – Definition of variables, and Supplementary Data Sheet 2 – Calculation of risk-adjusted mortality).

#### Measures for Intervention Processes and Implementation

To assess fidelity and extent of the local implementation of interventions in the participating hospitals, local quality improvement team leaders were surveyed in the autumns of the years 2016, 2017, and 2018. The survey used a standardized online questionnaire, which contained items on the status of existing quality management structures, extent of usage of quality analysis and implementation of recommended interventions, as well as perceived barriers to change, and rating of the support provided in the GQNS. Items of this questionnaire were designed based on results of qualitative interviews among quality improvement leaders during the MEDUSA study, a cluster-randomized controlled trial on a multifaceted educational intervention to improve acute sepsis care (10, 11).

#### Statistical Analysis

Retrospective baseline (January 2014–March 2016) and intervention phase (April 2016–June 2018) were descriptively compared regarding patients’ demographics, risk factors, the proportion of cases with mechanical ventilation >24 h, hospital length-of-stay, and mortality. The quarterly prevalence and RSMR were calculated and plotted to descriptively compare GQNS and the national DRG-statistics. To test the intervention effect, controlled interrupted time series analyses were calculated for each outcome (24). In this analysis each participating hospital provided its individual time series of monthly RSMRs. To incorporate the control condition, the difference between each monthly RSMR of each hospital and the RSMR obtained from the national DRG-statistics for this month was calculated. This defined a new time series for each hospital, representing the difference of its monthly RSMRs to the respective population value. The overall time series analysis incorporating this information from all hospitals was calculated by a piecewise hierarchical model (25). The intervention effect was then tested by the significance of the change in the linear slope as well as the significance of the change in level. Since small sample sizes of sepsis cases per month and hospital might cause bias by the unreliability of the RSMR estimate, the inverse of the noise-variance (see Supplementary Data Sheet 2) of the RSMR were used as precision weights in a sensitivity analysis.

Subgroup analyses were conducted among hospitals, which participated through the complete intervention phase, hospitals without complete participation, hospitals with ≤700 beds, and hospitals with >700 beds. Among the hospitals, which participated through the whole intervention period, a subgroup of hospitals was identified, which reported an early implementation of a sepsis-related quality management. This was defined, by the reporting of having implemented a quality improvement team as well as analyses of quality reports in the survey of quality improvement leaders in autumn of 2016.

To analyze the overall success of implementation of interventions as well as barriers and facilitators to change, descriptive statistics were calculated on the items of the last survey of quality improvement leaders – conducted in autumn of 2018. All analyses were conducted using the statistical software R, version 3.6.1 (26).

### Changes in the Evaluation Concept as Compared to the Study Protocol

The strategy of the evaluation was changed in some minor points. First, the primary analysis was not conducted as a difference-in-differences analysis but by a controlled interrupted time-series calculated using piecewise hierarchical models. This allowed to use all available information from all hospitals, regardless from when they joined the intervention, while at the same time controlling for seasonal variation and history bias. Second, the primary analysis was based on all hospitals participating in the GQNS, not only the hospitals participating from the beginning. Third, since the new sepsis-3 definitions do not include a sepsis without organ dysfunction anymore, no analysis was conducted for cases without coding of ICD-10-GM codes R65.1 or R57.2.

## Results

**FIGURE 1:**
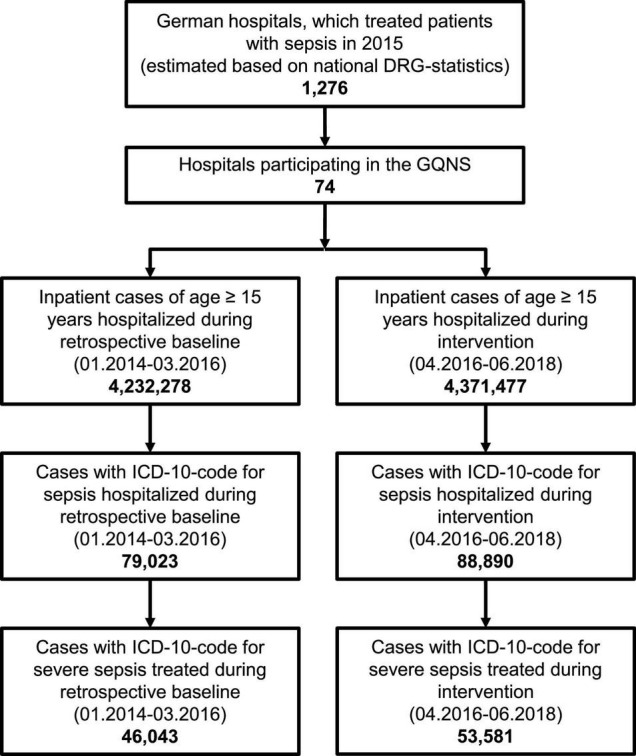
Study flow chart.

Forty-six hospitals received the first quality reports in April 2016, 28 additional hospitals joined later during the intervention phase. The participating 74 hospitals represent 5.7% of 1,276 German hospitals, which treated patients with sepsis (estimated based on national DRG-statistics of 2015). Figure 1 presents the flow chart of the inclusion of hospitals and cases. Characteristics of participating hospitals are presented in Supplementary Data Sheet 3 – Supplementary Table 1.

Characteristics of cases with sepsis are presented in Table 1. There were no relevant changes in demographics, comorbidities, or characteristics of the infection and sepsis. Hospital mortality was 43.5% during the retrospective baseline and 42.7% during the intervention period.

**TABLE 1:** Characteristics of included cases with coded sepsis.

Variable	Retrospective baseline (01.2014–03.2016)	Intervention phase (04.2016–06.2018)
Number of cases with coded sepsis	46.043	53.581
Age (years)	72 (60, 79)	72 (61, 79)
Sex: female	39%	38.7%
Admission: referral by physician or dentist	21.1%	19%
Emergency	63.7%	65.2%
Hospital transfer with pre-treatment >24 h	10.9%	11.3%
Hospital transfer with pre-treatment <24 h or rehabilitation hospital	4.3%	4.5%
**Comorbidities**		
CCI: cerebrovascular disease	12.8%	13.9%
CCI: dementia	8.5%	8.5%
CCI: mild liver disease	9.7%	10.1%
CCI: moderate or severe liver disease	4.2%	4.1%
CCI: myocardial infarction	10.5%	10.9%
CCI: peptic ulcer disease	4%	4.1%
ECI: alcohol abuse	7.1%	7.1%
ECI: blood loss anemia	0.9%	1%
ECI: cardiac arrhythmias	42.6%	44.7%
ECI: coagulopathy	39.3%	37.4%
ECI: congestive heart failure	34.4%	34.8%
ECI: deficiency anemia	4.4%	4.8%
ECI: depression	6%	5.9%
ECI: drug abuse	1.5%	1.8%
ECI: hypertension, complicated	10.1%	10.7%
ECI: hypertension, uncomplicated	42.2%	42.6%
ECI: hypothyroidism	11.6%	13.2%
ECI: lymphoma	3.5%	3.4%
ECI: metastatic cancer	7.6%	7.7%
ECI: obesity	9.1%	9.7%
ECI: other neurological disorders	15.6%	16.7%
ECI: paralysis	9.2%	9.8%
ECI: peripheral vascular disorders	16.6%	16.5%
ECI: psychoses	1.2%	1.1%
ECI: pulmonary circulation disorders	7.8%	8.1%
ECI: renal failure	30.2%	30.9%
ECI: solid tumor without metastasis	15.2%	14.6%
ECI: valvular disease	13%	14.4%
ECI: weight loss	11.6%	13.5%
Leukemia	3.8%	3.5%
**Characteristics of infection and sepsis**		
Infection of lower respiratory tract	48.5%	49%
Urinary tract infection	29.2%	30.9%
Abdominal infection	21.8%	20.3%
Foreign body associated infection	12.9%	12.6%
Soft tissue and wound infections	7.3%	8%
Infection of vascular system	5.6%	6%
Infection of central nervous system	1.9%	2.2%
Infection of upper respiratory tract	1.7%	2.9%
Sepsis as primary diagnosis	35.2%	33.4%
Conduction of chemotherapy	6.2%	6.4%
Conduction of palliative care	2.1%	2.1%
Hospital length of stay (days)	17 (8, 33)	16 (8, 31)
Hospital mortality	43.5%	42.7%

*Descriptive statistics presented as median (first quartile, third quartile) or %. CCI, Charlson comorbidity index; ECI, Elixhauser comorbidity index. Cases with sepsis defined by presence of ICD-10-GM codes R65.1 (sepsis with organ dysfunction) or R57.2 (septic shock). The beginning of the intervention phase is defined uniformly by April 2016 for all hospitals.*

### Tests of the Effect of Participation in the German Quality Network Sepsis

**FIGURE 2:**
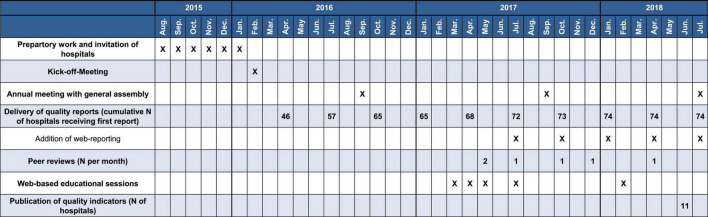
Time-line diagram on the progress of the GQNS.

**FIGURE 3:**
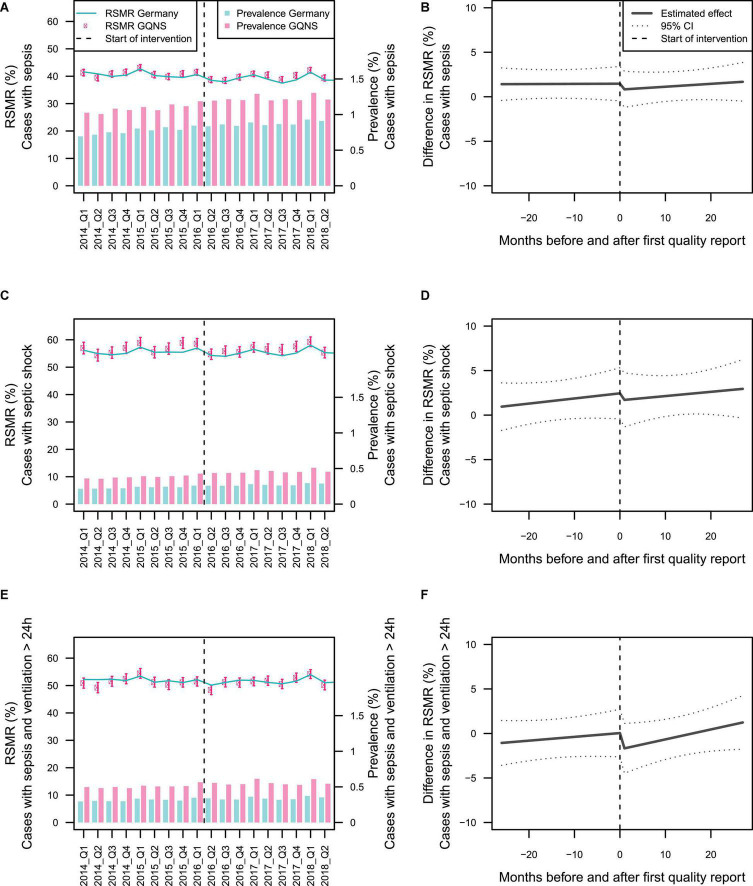
Depiction of the effect of hospitals’ participation in the GQNS. Panels **(A,C,E)** present the descriptive changes in prevalence and risk-standardized mortality rate (RSMR) for patients with sepsis, septic shock, and sepsis with mechanical ventilation >24 h. The beginning of the intervention phase is defined uniformly by April 2016 for all hospitals. Panels **(B,D,F)** depict the slopes before and after the beginning of the intervention, as well as the change in level at the beginning of the intervention with 95% prediction limits as estimated from interrupted time series analyses on the monthly RSMR-difference between GQNS hospitals and the national DRG-statistics. The beginning of the intervention phase is defined individually for each hospital by the date the first quality reports were provided to this hospital.

Figure 2 presents the time-line diagram of the progress of the GQNS. The results of the interrupted time series analysis on the difference between the RSMR of GQNS-hospitals and the RSMR from the national DRG statistics are presented in Table 2. There was no change in the trajectory of mortality for cases with sepsis across time before and after the intervention [percent change per month: 0.002 (95% CI: −0.074, 0.078), and 0.033 (−0.069, 0.134), respectively, test of difference: *p* = 0.632], and no significant change in level at the beginning of the intervention [percent change: −0.667 (−2.659, 1.324), *p* = 0.512]. This indicates that participation in the GQNS did not affect risk-adjusted mortality compared to the national DRG-statistics. Figure 3A presents the descriptive course of prevalence and RSMR for sepsis before and during the intervention period comparing participating hospitals in the GQNS and the national DRG-statistics; Figure 3B depicts the slopes and change in level calculated from the time series analysis.

**TABLE 2:** Results of interrupted time-series analyses on risk-standardized mortality rate difference between GQNS hospitals and the national diagnosis-related groups statistics.

Analysis	Number of hospitals	Slope before intervention (95% CI)	Slope during intervention (95% CI)	*P*-value of test of difference in slopes	Change in level (95% CI)	*P*-value
RSMR-difference for sepsis	74	0.002 (−0.074, 0.078)	0.033 (−0.069, 0.134)	0.632	−0.667 (−2.659, 1.324)	0.512
RSMR-difference for septic shock	74	0.058 (−0.073, 0.188)	0.048 (−0.123, 0.218)	0.928	−0.783 (−4.17, 2.603)	0.65
RSMR-difference for sepsis and mechanical ventilation >24 h	74	0.043 (−0.066, 0.152)	0.112 (−0.032, 0.256)	0.447	−1.827 (−4.669, 1.015)	0.208

*Results of piecewise hierarchical models on the difference in the risk-standardized mortality rate (RSMR) between GQNS hospitals and the national German diagnosis-related-groups statistic. Slopes give the linear trajectory of RSMR-difference in % per month across time before and after start of the intervention, change in level gives the change at the time of the beginning of the intervention. Time of beginning of the intervention is defined for each individual hospital as the time of supply of the first quality report.*

There were also no significant differences in slopes or changes in level in mortality among patients with septic shock or mortality among patients with sepsis and mechanical ventilation >24 h (Table 2 and Figures 3C–F). When precision weights were included to adjust for the unreliability of the RSMR estimated from small sample sizes, there were also no significant effects (data not shown).

When subgroups of hospitals were analyzed, there was a significant change in slopes from baseline to intervention for hospitals, which participated through the whole intervention (*p* = 0.042, Table 3). While the RSMR-difference showed a small increase during the baseline, there was no change across time observed anymore during the intervention, but there was also no decrease of mortality. No other subgroup showed any significant differences in slopes or level.

**TABLE 3:** Results of interrupted time-series analysis in subgroups of participating hospitals.

Subgroups	Number of hospitals	Slope before intervention (95% CI)	Slope during intervention (95% CI)	*P*-value of test of difference in slopes	Change in level (95% CI)	*P*-value
Participating through complete intervention period	45	0.133 (0.03, 0.236)	−0.018 (−0.12, 0.085)	0.042	−1.133 (−3.405, 1.138)	0.328
Participating through complete intervention period and early implementation of quality management*^a^*	8	−0.089 (−0.345, 0.167)	−0.035 (−0.29, 0.22)	0.771	2.841 (−2.784, 8.466)	0.323
Not participating through complete intervention period	29	−0.076 (−0.193, 0.041)	0.165 (−0.085, 0.415)	0.084	−1.67 (−5.525, 2.184)	0.396
Number of beds ≤700	40	0.017 (−0.117, 0.152)	0.02 (−0.153, 0.194)	0.98	−0.997 (−4.468, 2.474)	0.573
Number of beds >700	34	−0.015 (−0.073, 0.042)	0.047 (−0.033, 0.127)	0.21	−0.285 (−1.828, 1.257)	0.717

*Results of piecewise hierarchical models on the difference in the risk-standardized mortality rate (RSMR) in patients with sepsis between GQNS hospitals and the national German diagnosis-related-groups statistic considering different subgroups. Slopes give the linear trajectory of RSMR-difference in % per month across time before and after start of the intervention, change in level gives the change at the time of the beginning of the intervention. Time of beginning of the intervention is defined for each individual hospital as the time of supply of the first quality report. ^a^Early implementation of quality management was defined based on the survey of local quality improvement leaders of participating hospitals in autumn of 2016, if the implementation of a quality improvement team as well as analyses of quality reports was reported. Survey data were available for 28 of 45 hospitals (62%).*

### Process Evaluation

Table 4 presents the survey results among local quality improvement leaders, 49 of 69 (71%) invited participants took part in the survey. The results show an overall low degree of implementation of quality management processes: only 22 (44.9%) of hospitals did a complete analysis of provided information on the quality of care by using both the comparison of quality indicators as well as individual case analysis, only eight (16.3%) had an interdisciplinary quality improvement team. Likewise, the implementation degree of measures to improve early recognition, and adequate treatment of sepsis was low: in half of the hospitals, there was no regular staff education on sepsis in the emergency department [*N* = 23 (46.9%)], and on normal wards [*N* = 25 (52.1%)]. Medical emergency teams were implemented in only eight (16.3%) of surveyed hospitals; only three hospitals (6.1%) had screening tools for early detection of sepsis in all relevant departments. Local quality improvement leaders reported high barriers to quality improvement efforts. The GQNS was not seen as an important quality measure for the complete hospital in most hospitals. The most important barriers were lack of time of the quality improvement team [*N* = 38 (77.6%)], general staff shortage [*N* = 29 (59.2%)], and lack of participation of relevant departments [*N* = 19 (38.8%)]. The overall rating of the support provided in the GQNS was good (median grade of 2 for work of the coordination bureau, as well as usefulness and usability of quality reports).

**TABLE 4:** Results of survey of the local quality improvement leaders of participating hospitals.

Items of the survey	Descriptive statistics for answers (*N* = 49 participants)
**Implementation of quality improvement measures**	
Usage of quality reports	
None received yet/unknown	6(12.2%)
Not used yet	7(14.3%)
Quality indicators analyzed	14(28.6%)
Quality indicators and individual cases analyzed	22(44.9%)
Existence of a quality improvement team	
No	33(67.3%)
Yes, but not interdisciplinary	8(16.3%)
Yes, interprofessional and interdisciplinary	8(16.3%)
Staff education on ICU	
No or unknown	7(14.3%)
Partly implemented	25(51%)
Fully implemented	17(34.7%)
Staff education in emergency department	
No or unknown	23(46.9%)
Partly implemented	15(30.6%)
Fully implemented	11(22.4%)
Staff education on normal wards*^a^*	
No or unknown	25(52.1%)
Partly implemented	19(39.6%)
Fully implemented	4(8.3%)
Implementation of screening tools	
Not implemented	19(38.8%)
Implemented on ICU	8(16.3%)
Implemented in at least one other department	19(38.8%)
Implemented on ICU, normal wards, and emergency department	3(6.1%)
Existence of medical emergency team	
Not planned	24(49%)
Planned	17(34.7%)
Existing	8(16.3%)
**Barriers to implementation of quality improvement**	
Importance of GQNS for the hospital	
No importance	14(28.6%)
One among many quality improvement measures	17(34.7%)
Important in some departments	13(26.5%)
Important for the complete hospital	5(10.2%)
Lack of time of quality improvement team	38(77.6%)
General staff shortage	29(59.2%)
Lacking participation of relevant departments	19(38.8%)
Tribal thinking of departments	12(24.5%)
Lacking decision making power of responsible team	10(20.4%)
Lacking support by management	8(16.3%)
Lacking awareness of the need for quality improvement	5(10.2%)
Strict management-hierarchy	4(8.2%)
**Rating of the support by the GQNS**	
Grade for the work of the GQNS coordination bureau (1–6)	2(1,2)
Grade for usefulness of quality reports (1–6)	2(1,2)
Grade for usability of quality reports (1–6)	2(2,2)

*Descriptive statistics given as N (%) and median (first quartile, third quartile). The survey was conducted among the local quality improvement leaders of participating hospitals in autumn of 2018 after the end of the intervention phase, one person per hospital was surveyed, since some local champions were responsible for more than 1 hospital, 69 participants were invited of which, 49 (71%) took part in the survey. ^a^One participant did not provide information on this item.*

## Discussion

The GQNS is a quality collaborative network using claims data and a complex risk adjustment to measure and improve the acute care quality for sepsis patients. Because of this pragmatic approach, 74 hospitals participated in the start-up period of the network. This evaluation study compared the development of risk-adjusted hospital mortality in cases with sepsis between the GQNS and the German national DRG-statistics in a controlled time series analysis. It did not show an effect of participation in the GQNS.

The failure to achieve substantial improvement might be caused by specific flaws in the approach taken by the GQNS. First, the GQNS only measured outcome quality in the form of risk-adjusted sepsis mortality, which alone does not give detailed insights into concrete possibly underlying care deficiencies (27). Former successful quality initiatives on sepsis also used process quality indicators – primarily compliance to sepsis management bundles like timeliness of adequate antimicrobial therapy (9, 28–31). Additionally, benchmarking indicators of structural quality – like availability of in-house microbiological or standard operating procedures on antimicrobial treatment – could inform hospitals to implement concrete improvements. Second, the GQNS relied on only using administrative claims data. This approach has high feasibility and low costs, but lacks the information necessary to define process quality indicators. Above that, identification of cases based on ICD-coding in administrative data can be impaired by a misclassification bias (32). Several studies reported low sensitivity for coding of sepsis (33, 34). Misclassification also explains the high observed sepsis-related mortality of more than 40%, since studies have shown that patients with higher risk of death have a higher probability of having an explicit sepsis code in administrative data (34, 35). Also risk factors for mortality – like comorbidities – have been shown to be subject to misclassification (36). The low validity of the data might have impaired the usefulness of the quality reports to identify possible deficiencies of care and opportunities for improvement (12). Automated surveillance may overcome these deficits to track sepsis rates and outcomes based on electronic health records (37), but cannot currently be used among the majority of German hospitals due to the lack of implementation of electronic health records. Third, the only mandatory elements of the intervention were reporting, benchmarking, and publication of quality indicators. Hospitals were advised to form interdisciplinary quality improvement teams to establish a continuous quality improvement based on the analysis of the data provided in the quality reports, case analysis, and peer-reviews. This approach might not have been sufficient to achieve substantial changes, since hospitals have repeatedly been shown to have major deficiencies in organizational and professional capacity to adequately learn and improve based on quality measurement (10, 38, 39), Therefore, implementing a core set of well-defined interlinked improvement measures, like hospital-wide staff education on early recognition and treatment, regular screenings on wards and in emergency departments, and medical emergency teams, in all participating hospitals using a well-structured implementation strategy, could be more successful (9, 28, 29, 40).

The major reason for the failure of the GQNS to achieve a reduction of sepsis-related mortality can be seen in the lack of implementation of measures for quality improvement by the majority of hospitals. Local quality improvement leaders reported high barriers to effective quality management – most importantly, lack of time and resources for quality improvement activities, as well as failure to generate hospital-wide improvement efforts due to general staff shortage and lack of involvement of all relevant departments and stakeholders. Similar reasons had been identified for the failure of the cluster-randomized controlled MEDUSA trial, which comprised 40 German hospitals and aimed to improve sepsis care by the establishment of change teams and prospective documentation and reporting of indicators of process and outcome quality, and staff education (10, 11). Likewise, the only published successful quality initiative on sepsis in Germany, by which an absolute reduction of mortality of 19% was achieved, received financing and full support by the hospital’s management board, which facilitated the hospital-wide role out of this program and the involvement of the crucial stakeholders (31).

The failure to replicate such successes in multicenter initiatives like the GQNS and MEDUSA point to the limitations of voluntary quality initiatives, which may often not be able to achieve adequate priority among hospital management boards and department leaderships. Sepsis-specific mandatory quality improvement indicators and tools have been implemented on the national and regional levels in several countries and were associated with decreased sepsis-related mortality (30, 37, 40, 41). Care processes for patients with sepsis are also affected by more general tools for quality assurance and patient safety – such as rapid response systems, nation-wide education of health care workers in early warning scores for deteriorating patients, and the effective use of critical incidence reporting systems. These are mostly standard in other high-income countries like the United Kingdom, Australia or in part the United States, but are poorly adopted in Germany (42–46). German authorities and regulatory bodies in health care should follow these examples, become fully aware of the existing severe deficits in sepsis prevention and care, and take the necessary actions. An essential step would be the inclusion of indicators on the quality of acute sepsis care to the mandated quality assurance system for hospitals in Germany (47). These indicators should include aspects of structural quality – like regular education of all clinical staff on early detection and treatment of sepsis, aspects of process quality – like implementation of a standardized screening for patients at risk (40), and documentation of adequacy of implementation of guideline elements (9, 31), as well as outcome quality – like risk-adjusted mortality and morbidity of survivors (48).

### Strengths and Limitations

The evaluation study of GQNS has several strengths. Because of its controlled interrupted time series design, it has higher internal validity compared to most previous evaluation studies on sepsis-related quality initiatives, which only used before-after comparisons (9). In addition, a diverse sample representing the full spectrum of German acute care hospitals was included, which permits generalizing conclusions to the German health care system. The evaluation study also has limitations. It was based on claims data and might therefore be biased by changes in coding practices among participating hospitals across time. Although new clinical sepsis definitions (“sepsis-3”) were introduced in 2016 (1), the ICD-coding of sepsis relied on the old sepsis-1 definitions until the end of 2019 in Germany, which might influence the generalization of the results of this study. The national DRG-statistics, which were used as control condition, also included the data of the hospitals participating in the GQNS. This reduced the effect size of possible differences between GQNS and the national statistics, but we believe this bias to be small, since the GQNS-hospitals represent only 6% of all German hospitals. Process evaluation was only based on yearly standardized surveys of local quality improvement leaders and not all hospitals provided this data. A more frequent assessment and report of the implementation progress could have helped to motivate stakeholders of participating hospitals to increase their efforts. The duration of the intervention phase of roughly 2 years might have been too short to result in observable changes (49). We were only able to conduct six peer reviews during the intervention phase of the GQNS, since the number of qualified peers was limited and finding appointments was complicated due to the busy schedules of involved clinicians. To overcome this problem, education of peer reviewers was established in 2020 within the GQNS. The first publication of the main quality indicators occurred in the summer of 2018, at the end of the intervention phase, and only by the 11 hospitals, which were obligated to do so since they had signed their contract for participation in 2015. It is unclear if a broader early implementation of these core elements of the intervention would have resulted in greater success.

## Conclusion

Participation in this voluntary quality initiative did not result in a reduction of sepsis-related hospital mortality. Major barriers to quality improvement were lack of time and resources for quality improvement teams, general staff shortage, and a failure to involve all relevant stakeholders and departments in the quality improvement process. Voluntary quality initiatives may not be able to achieve adequate priority among pertinent stakeholders among hospital board and department leadership. Therefore, sepsis needs to become part of the mandated external quality assurance for all German hospitals to end preventable suffering from sepsis and reduce the burden for the German health care system.

## Data Availability Statement

The datasets presented in this article are not readily available because by contract with the participating hospitals of the GQNS, the research team is not allowed to publish data, which would make individual hospitals identifiable by third parties. Requests to access the datasets should be directed to DS, Daniel.Schwarzkopf@med.uni-jena.de.

## Ethics Statement

The studies involving human participants were reviewed and approved by the Internal Review Board of the Jena University Hospital. Written informed consent from the participants or their legal guardian/next of kin was not required to participate in this study in accordance with the national legislation and the institutional requirements.

## Members of the GQNS Study Group

Katrin Baete, Gesundheit Nord gGmbH, Germany; Timm Bauer, Sana Klinikum Offenbach GmbH, Germany; Peter Brand, KMG Klinikum Sömmerda, Germany; Josef Briegel, Klinikum der Universität München, Germany; Udo Brüderlein, Klinikum Südstadt Rostock, Germany; Michael Bucher, Universitätsklinikum Halle (Saale), Germany; Karin Dey, Bundeswehrkrankenhaus Berlin, Germany; Anja Diers, Klinikum Oldenburg AöR, Germany; Norbert Dietrich, DRK-Krankenhaus Mecklenburg-Strelitz gGmbH, Germany; Holding Doris, Asklepios Kliniken Hamburg GmbH – Asklepios Klinik Nord-Heidberg, Germany; Michael Ebenhoch, Berufsgenossenschaftliche Unfallklinik Murnau (BGU), Germany; Beatrix Eberhardt, Südharz Klinikum Nordhausen gemeinnützige GmbH, Germany; Thomas Eberle, MediClin Herzzentrum Coswig, Germany; Leila Eckholt, Vivantes Klinikum Am Urban, Germany; Heidrun Ehmcke, MediClin Müritz-Klinikum, Germany; Fritz Fiedler, St. Elisabeth-Krankenhaus GmbH, Germany; Martin Franz, Vivantes Klinikum Kaulsdorf, Germany; Reiner Giebler, Diakonie-Klinikum GmbH, Germany; Jürgen Götz, Klinikum Lippe GmbH, Germany; Heinrich V. Groesdonk, HELIOS Klinikum Erfurt, Germany; Yvonne Hartmann, AMEOS Klinikum Ueckermünde, Germany; Rolf Hauschild, St. Georg Klinikum Eisenach gemeinnützige GmbH, Germany; Markus Heim, Klinikum rechts der Isar der Technischen Universität München, Germany; Bernhard Heising, Krankenhaus Düren gem. GmbH, Germany; Oliver Herden-Kirchhoff, AMEOS Klinikum Bremerhaven GmbH, Germany; Peter Hilbert-Carius, BG Klinikum Bergmannstrost Halle gGmbH, Germany; Ulrich Jaschinski, Universitätsklinikum Augsburg, Germany; Stefan John, Klinikum Nürnberg, Germany; Veit Kinne, Universitätsklinikum Jena, Germany; Martin Kocur, DRK Manniske Krankenhaus Bad Frankenhausen, Germany; Klaus Kogelmann, Klinikum Emden – Hans-Susemihl-Krankenhaus gemeinnützige GmbH, Germany; Jens-Peter König, Sana Klinikum Lichtenberg, Germany; Heinrich Rudolf Kosiek, AMEOS Klinikum Alfeld GmbH, Germany; Anja Kubiessa, Pleißental-Klinik GmbH, Germany; Martina Lange, Waldkrankenhaus “Rudolf Elle” GmbH, Germany; Peter Lehmkuhl, Vivantes Auguste-Viktoria-Klinikum, Germany; Stefan Lenz, Havelland Kliniken GmbH, Germany; Oliver Mayer, Universitätsklinikum Ulm, Germany; Toralf Morgenstern, Städtisches Klinikum Dresden, Germany; Ralf Michael Muellenbach, Klinikum Kassel GmbH, Germany; Carla Nau, Universitätsklinikum Schleswig-Holstein Campus Lübeck, Germany; Lorenz Nowak, Asklepios Klinik Gauting GmbH, Germany; Nina Polze, Universitätsklinikum Leipzig, Germany; Karsten Pracht, Sana Kliniken Leipziger Land GmbH Standort Borna, Germany; Christian Putensen, Universitätsklinikum Bonn, Germany; Yvonne Rakow, Medizinische Hochschule Hannover (MHH), Germany; Frank Reichel, KMG Klinikum Sondershausen, Germany; Thomas Reinz, SRH Waldklinikum Gera GmbH, Germany; Daniel Reuter, Universitätsmedizin Rostock – Rechtsfähige Teilkörperschaft der Universität Rostock, Germany; Matthias Richl, Kreiskliniken des Landkreises Mühldorf a. Inn GmbH, Germany; Gabriella Rimkus, Universitätsklinikum Jena, Germany; Thomas Rohark, Universitätsklinikum Düsseldorf, Germany; Peter Rosenberger, Universitätsklinikum Tübingen, Germany; Winfried Ruf, Vivantes Klinikum Spandau, Germany; Axel Schipp, DRK-Krankenhaus Grimmen GmbH, Germany; Rolf-Jürgen Schröder, AMEOS Klinikum Ueckermünde, Germany; Konrad Schwarzkopf, Klinikum Saarbrücken gGmbH, Germany; Rüdiger Sinz, Asklepios-ASB Krankenhaus Radeberg GmbH, Germany; Ivan Tanev, Universitätsklinikum Magdeburg, Germany; Rolf Teßmann, Berufsgenossenschaftliche Unfallklinik Frankfurt am Main gGmbH, Germany; Manfred Thiel, Universitätsklinikum Mannheim, Germany; Ulrich Treichel, Asklepios Kliniken Schildautal – Asklepios Klinik Sobernheim GmbH, Germany; Katrin Umgelter, Vivantes Humboldt-Klinikum, Germany; Gebhard von Cossel, Sana Kliniken AG, Germany; Christian von Heymann, Vivantes Klinikum im Friedrichshain – Landsberger Allee, Germany; Norbert Weiler, Universitätsklinikum Schleswig-Holstein Campus Kiel, Germany; Harald Weng, Klinikum der Landeshauptstadt Stuttgart, Germany; Maria Zach, Kreiskrankenhaus Wolgast gGmbH, Germany; Kai Zacharowski, Universitätsklinikum Frankfurt, Germany; Kristin Zapf, SRH Zentralklinikum Suhl GmbH, Germany.

## Author Contributions

KR conceptualized the study design, acquired the funding for the study, and supervised the conduction of the study. DS and HR contributed to the coordination of the GQNS, the conduction of the interventions, and the acquisition of the reported data. DS conceptualized the quality reporting, conducted the analyses, and drafted the manuscript. CF-S, and DT-R contributed to the development of the methods for the quality reporting. AB, CG, MGl, MGr, PM, MP, and TS were involved in the supervision of the conduction of the GQNS as members of the steering-committee. KR, HR, AB, CF-S, DT-R, CG, MF, MGl, MGr, PM, MP, and TS contributed to the interpretation of the data and critically revised the manuscript. All authors read and approved the final manuscript.

## Conflict of Interest

DS and HR were funded in part by grants from the German Federal Ministry of Education and Research during and outside the submitted work and by annual fees paid by hospitals to participate in the GQNS. CF-S was funded by grants from the German Federal Ministry of Education and Research and the German Innovations Fund of the Federal Joint Committee in Germany (G-BA), outside the submitted work. MGr reports grants from German Federal Ministry of Education and Research, outside the submitted work. MP reports grants by the Federal Ministry of Education and Research, outside the submitted work. DT-R reports grants from German Federal Ministry of Education and Research, outside the submitted work. KR was shareholder with less of 0.5% of InflaRx NV a Jena/Germany based Biotech Company that evaluates a immunmodulatory approach for the adjunctive treatment of COVID-19. MGl was employed by KH Labor GmbH. The remaining authors declare that the research was conducted in the absence of any commercial or financial relationships that could be construed as a potential conflict of interest.

## Publisher’s Note

All claims expressed in this article are solely those of the authors and do not necessarily represent those of their affiliated organizations, or those of the publisher, the editors and the reviewers. Any product that may be evaluated in this article, or claim that may be made by its manufacturer, is not guaranteed or endorsed by the publisher.

## Abbreviations

DRG: diagnosis-related groups
GQNS: German Quality Network Sepsis
ICD: International Classification of Diseases
ICD-10-GM: International Statistical Classification of Diseases and Related Health Problems – German Modification – 10th Revision
RSMR: risk standardized mortality rate
USA: United States of America.
