# Supplementary material for: The German Quality Network Sepsis: Evaluation of a Quality Collaborative on Decreasing Sepsis-Related Mortality in a Controlled Interrupted Time Series Analysis
Source: Front Med (Lausanne). 2022 Apr 27;9:882340. doi: 10.3389/fmed.2022.882340 (PMC9094049; doi:10.3389/fmed.2022.882340)
Supplement: Supplementary file 1 [file Data_Sheet_1.pdf]

# Supplementary Material 1: Definition of variables

## 1 Definitions of variables based on administrative claims data

| Variable | Definition |
|---|---|
| Patient demographics | |
| Gender | Coded "0" if male and "1" if female |
| Hospital admission | |
| Hospital admission type | Admission types in DRG data were collapsed because of small case numbers in types “Z” and “R”: 1. Emergency (type “N”); 2. Referral by physician or dentist (type “E” or “Z“); 3. Hospital transfer with pre-treatment >24h (type “V”); 4. Hospital transfer with pre-treatment < 24h or rehabilitation hospital (type “A” or “R”) |
| Clinical characteristics of the infection | |
| Septic shock | ICD-10 codes in primary or secondary diagnoses: R572 |
| Sepsis as primary diagnosis[a] | ICD-10 codes in primary diagnosis: A400; A401; A402; A403; A408; A409; A410; A411; A412; A413; A414; A4151; A4152; A4158; A418; A419; R572; R650; R651 |
| Infection of lower respiratory tract | ICD-10 codes in primary or secondary diagnoses: J12; J13; J14; J15; J16; J17; J18; J20; J21; J22; J440; J441; J47; J86; J85; A15; A16; U6900 |
| Infection of upper respiratory tract | ICD-10 codes in primary or secondary diagnoses: J09; J10; J11; J00; J01; J02; J03; J04; J06; J05; A36; A37 |
| Abdominal infection | ICD-10 codes in primary or secondary diagnoses: A00; A01; A02; A03; A04; A05; A06; A07; A08; A09; K35; K37; K36; K5712; K5702; K5713; K5703; K5722; K5732; K5723; K5733; K5742; K5743; K5752; K5753; K5782; K5783; K5792; K5793; K61; K65; K67; K630; K631; K750; K751; K810 |

| | |
|---|---|
| Soft tissue and wound infections | ICD-10 codes in primary or secondary diagnoses: A46; B35; B36; B47; L03; L04; L08; L88; L05; B00 |
| Urinary tract infection | ICD-10 codes in primary or secondary diagnoses: N10; N11; N12; N151; N159; N16; N288; N34; N30; N390; N41; N45; N51; N482; N49; N70; N71; N72; N73; N74; N75; N76; N77; N61 |
| Infection of central nervous system | ICD-10 codes in primary or secondary diagnoses: A39; G00; G01; G02; G03; G04; G05; G06; G07; G08; A17 |
| Infection of vascular system | ICD-10 codes in primary or secondary diagnoses: I30; I32; I33; I39; I40; I41; I80 |
| Foreign body associated infection | ICD-10 codes in primary or secondary diagnoses: T826; T827; T835; T836; T845; T846; T847; T857 |
| Comorbidities[b] | |
| CCI: Cerebrovascular disease | ICD-10 codes in primary or secondary diagnoses: G4589; G462; G4583; G4503; G4592; G4539; G4599; G460; G461; G4593; I600; G4542; G4543; G4523; G467; H340; I606; G4509; G4512; G4513; G4519; G4522; I612; I613; I601; I602; I603; I604; I605; G468; I607; I608; I609; I610; I611; I630; I631; G463; G464; G465; G466; I619; G4502; I639; I64; I650; I651; I652; I653; I658; I659; I660; I661; I662; I663; I664; I668; I669; I670; I6710; I6711; I672; I673; I674; I614; I615; I616; I618; I6788; I6200; I6201; I6202; I6209; I621; I629; I691; I692; I632; I633; I634; I635; I636; I638; I679; G4549; G4582; I682; I688; I690; G4533; I6780; G4529; G4532; I676; I681; I698; I680; I693; I677; I675; I694 |
| CCI: Dementia | ICD-10 codes in primary or secondary diagnoses: F012; F021; F011; F001; F03; F009; F010; F051; F013; F018; F019; F023; F000; F002; F022; F024; F028; G300; G301; G308; F020; G311; G309 |
| CCI: Mild liver disease | ICD-10 codes in primary or secondary diagnoses: B180; B181; B182; B188; B189; K746; K760; K762; K763; K764; K702; K703; K739; K768; K769; K713; K714; K715; K717; K732; K738; K700; K701; Z944; K742; K743; K709; K745; K741; K744; K731; K730; K740 |

| | |
|---|---|
| CCI: Moderate or severe liver disease | ICD-10 codes in primary or secondary diagnoses: K704; K711; K765; K767; I982; K729; K766; I864; I850; I859; I983; K721 |
| CCI: Myocardial infarction | ICD-10 codes in primary or secondary diagnoses: I211; I212; I229; I2522; I210; I219; I2529; I221; I213; I2521; I214; I220; I228; I2520 |
| CCI: Peptic ulcer disease | ICD-10 codes in primary or secondary diagnoses: K270; K266; K267; K289; K271; K272; K269; K264; K265; K250; K251; K252; K253; K254; K255; K256; K257; K259; K260; K261; K262; K263; K280; K281; K282; K283; K284; K285; K286; K273; K274; K275; K276; K277; K279; K287 |
| ECI: Alcohol abuse | ICD-10 codes in primary or secondary diagnoses: F108; K703; F104; T510; I426; K700; G621; F105; T518; T511; Z502; F109; T513; K709; F103; T512; F102; E52; F107; K292; F100; T519; F101; F106 |
| ECI: Blood loss anemia | ICD-10 codes in primary or secondary diagnoses: D500 |
| ECI: Cardiac arrhythmias | ICD-10 codes in primary or secondary diagnoses: I459; I495; I498; I442; I471; I443; I456; I492; I489; I494; Z950; I483; I470; I479; R001; R008; I499; I441; Z4508; I480; I490; I493; R000; Z4500; I481; I472; I491; Z450; Z4501; I484; I482; T821; Z4502 |
| ECI: Coagulopathy | ICD-10 codes in primary or secondary diagnoses: D6835; D686; D681; D6838; D6834; D691; D6832; D684; D685; D67; D680; D6952; D6833; D6821; D6824; D6825; D6820; D650; D651; D652; D659; D66; D6940; D6941; D6823; D6953; D6957; D6822; D6831; D6959; D6826; D6828; D6961; D693; D693; D6960; D6958; D689; D688 |
| ECI: Congestive heart failure | ICD-10 codes in primary or secondary diagnoses: I4288; I4280; I5019; I427; I509; I432; I438; I430; I431; P290; I099; I5012; I5013; I5014; I420; I425; I426; I5011; I5001; I429; I5000; I255 |
| ECI: Deficiency anemia | ICD-10 codes in primary or secondary diagnoses: D508; D512; D509; D520; D521; D528; D538; D510; D511; D529; D518; D513; D539; D532; D530; D519; D531 |
| ECI: Depression | ICD-10 codes in primary or secondary diagnoses: F323; F328; F314; F322; F204; F315; F329; F333; F321; F432; F313; F332; F330; F331; F338; F339; F320; F334; F412; F341 |

| | |
|---|---|
| ECI: Drug abuse | ICD-10 codes in primary or secondary diagnoses: F128; F122; F121; F134; F127; F133; F126; F131; F166; F167; F130; F160; F161; F162; F159; F125; F165; F138; F139; F129; F169; F180; F132; F144; F181; F182; F184; F135; F136; F123; F110; F111; F112; F113; F168; F115; F116; F145; F146; F147; F148; F149; F137; F164; F188; F189; F190; F114; F192; F193; F117; F118; F119; F120; F185; F124; F187; F150; F151; F152; F191; F154; F155; F194; F195; F196; F183; F198; F186; F163; F140; F141; F156; F153; F157; F197; F158; F143; F199; F142 |
| ECI: Hypertension, complicated | ICD-10 codes in primary or secondary diagnoses: I1101; I1101; I1191; I1200; I1320; I1310; I1100; I1501; I1301; I1320; I1310; I1190; I1311; I1300; I1321; I1301; I1100; I1510; I1511; I1520; I1311; I1580; I1390; I1200; I1290; I1291; I1201; I1300; I1500; I1521; I1591; I1581; I1201; I1321; I1391; I1590 |
| ECI: Hypertension, uncomplicated | ICD-10 codes in primary or secondary diagnoses: I1010; I1091; I1011; I1001; I1090; I1000 |
| ECI: Hypothyroidism | ICD-10 codes in primary or secondary diagnoses: E012; E018; E011; E033; E010; E030; E000; E038; E02; E039; E009; E031; E032; E890; E001; E002; E035; E034 |
| ECI: Lymphoma | ICD-10 codes in primary or secondary diagnoses: C845; C846; C848; C810; C849; C851; C847; C857; C817; C859; C852; C965; C966; C967; C968; C969; C826; C827; C829; C830; C831; C9030; C819; C820; C821; C822; C823; C824; C825; C864; C865; C811; C812; C813; C814; C9031; C960; C962; C964; C838; C841; C844; C8890; C8891; C9000; C9001; C9020; C9021; C8821; C8830; C8831; C860; C861; C862; C863; C840; C8801; C8820; C866; C8800; C8840; C8841; C8870; C833; C839; C835; C837; C8871 |
| ECI: Metastatic cancer | ICD-10 codes in primary or secondary diagnoses: C793; C797; C794; C7983; C788; C791; C792; C774; C775; C795; C790; C796; C783; C778; C779; C7981; C770; C771; C772; C773; C799; C800; C809; C784; C780; C781; C782; C7984; C785; C786; C787; C7982; C7988 |
| ECI: Obesity | ICD-10 codes in primary or secondary diagnoses: E6699; E6629; E6602; E6622; E6692; E6680; E6609; E6691; E6621; E6611; E6601; E6690; E6689; E6681; E6612; E6610; E6620; E6600; E6682; E6619 |

| | |
|---|---|
| ECI: Other neurological disorders | ICD-10 codes in primary or secondary diagnoses: G130; G934; G328; G350; G112; G131; G129; G931; G369; G319; G138; G121; G122; G132; G128; G210; G211; G119; G213; G214; G2000; G212; G118; G3521; G120; G3531; G359; G2091; G3188; G320; G10; G110; G111; G113; G2001; G2010; G3520; G375; G378; G379; G3530; G4001; G4002; G360; G361; G370; G371; G372; G373; G374; G406; G407; G408; G4000; G410; G411; G4008; G4009; G401; G402; G403; G404; G405; G218; G219; G22; G409; G2020; G2021; G3182; G412; G418; G419; R470; G3510; G3511; G114; G312; G2090; G2011; G255; G368; R560; R568; G3181; G254 |
| ECI: Paralysis | ICD-10 codes in primary or secondary diagnoses: G8212; G8266; G833; G8211; G8263; G8264; G8267; G831; G832; G8265; G8201; G8341; G8349; G8200; G041; G8261; G8233; G8239; G8240; G8213; G8219; G819; G830; G811; G8340; G8203; G8209; G839; G8210; G8260; G8262; G8221; G8241; G8242; G114; G8229; G8222; G8250; G8202; G8220; G8230; G8232; G802; G8223; G8269; G810; G8231; G8259; G8252; G8253; G8249; G801; G8251; G8243 |
| ECI: Peripheral vascular disorders | ICD-10 codes in primary or secondary diagnoses: I7021; I7020; I7103; I7104; I738; I7023; I7102; I731; I792; I739; I771; I790; I709; I7100; I7101; I719; I701; I700; I7024; I7105; I7022; I7107; Z9580; I7025; I708; I7029; I714; Z9581; Z9588; Z959; K559; I711; I712; I7106; I713; I715; I716; I7026; K5588; K551; K5581; I718; K5582 |
| ECI: Psychoses | ICD-10 codes in primary or secondary diagnoses: F24; F239; F220; F238; F232; F230; F233; F251; F252; F202; F203; F259; F208; F209; F28; F200; F231; F312; F228; F258; F250; F204; F205; F315; F29; F302; F201; F229; F206 |
| ECI: Pulmonary circulation disorders | ICD-10 codes in primary or secondary diagnoses: I288; I2720; I2728; I280; I278; I260; I269; I271; I270; I289; I279 |
| ECI: Renal failure | ICD-10 codes in primary or secondary diagnoses: N250; Z490; Z491; Z492; N19; Z940; N181; N182; N183; N184; N185; N1880; N1889; N189; Z992 |
| ECI: Solid tumor without metastasis | ICD-10 codes in primary or secondary diagnoses: C07; C080; C068; C069; C161; C154; C155; C158; C159; C160; C248; C081; C088; C089; C090; C166; C091; C169; C001; C002; C003; C004; C005; C006; C162; C163; C164; C165; C021; C022; C023; C024; C028; C029; C030; C031; C039; C040; C041; C048; C049; C221; |

| | |
|---|---|
| | C222; C223; C224; C227; C229; C061; C062; C383; C384; C388; C390; C398; C399; C400; C168; C000; C098; C099; C100; C101; C102; C103; C008; C009; C01; C020; C111; C112; C113; C118; C119; C12; C130; C131; C132; C138; C139; C140; C142; C148; C150; C151; C152; C153; C460; C461; C462; C463; C467; C468; C469; C470; C471; C472; C401; C402; C475; C170; C171; C172; C173; C178; C104; C108; C109; C110; C183; C184; C185; C186; C187; C188; C189; C19; C20; C210; C211; C212; C218; C220; C508; C509; C510; C511; C512; C518; C519; C52; C530; C531; C538; C539; C540; C541; C473; C474; C548; C549; C55; C56; C570; C571; C572; C573; C574; C577; C578; C579; C58; C600; C601; C602; C608; C609; C61; C620; C621; C629; C630; C631; C632; C637; C638; C639; C64; C65; C66; C670; C671; C672; C673; C674; C675; C676; C677; C678; C679; C403; C408; C409; C4101; C4102; C411; C412; C4130; C4131; C4132; C414; C418; C419; C430; C431; C432; C433; C434; C435; C436; C437; C438; C439; C450; C451; C452; C457; C459; C722; C723; C724; C725; C728; C729; C73; C740; C741; C749; C750; C751; C752; C753; C754; C755; C758; C759; C760; C761; C762; C763; C764; C765; C767; C768; C495; C496; C498; C499; C500; C501; C502; C503; C504; C505; C506; C050; C051; C052; C058; C059; C060; C23; C240; C241; C269; C249; C250; C251; C542; C543; C254; C257; C258; C259; C260; C261; C268; C328; C300; C301; C310; C311; C312; C313; C318; C319; C320; C321; C322; C323; C690; C329; C33; C340; C341; C717; C718; C719; C720; C721; C37; C381; C382; C711; C691; C97; C693; C694; C695; C696; C252; C253; C680; C681; C688; C689; C479; C712; C692; C714; C715; C716; C182; C698; C699; C700; C701; C709; C710; C180; C480; C713; C179; C343; C181; C488; C342; C380; C348; C349; C476; C492; C493; C482; C481; C478; C494; C490; C491 |
| ECI: Valvular disease | ICD-10 codes in primary or secondary diagnoses: A520; I3480; I3488; I349; I350; I351; Z952; Z953; Z954; I360; I361; I340; I341; I342; I370; I371; I372; I378; I379; I352; I358; I359; I392; I393; I362; I368; I369; I051; I052; I058; I059; Q230; I38; I390; I391; I069; I070; I394; I398; I050; I079; I080; I081; I082; I083; Q231; Q232; Q233; I098; I068; I071; I072; I078; I062; I088; I089; I091; I061; I060 |
| Leucemia | ICD-10 codes in primary or secondary diagnoses: C901; C91; C92; C93; C94; C95 |
| Specific procedures | |

| | |
|---|---|
| Conduction of palliatve care | German procedure codes: 8982; 898e |
| Conduction of chemotherapy | German procedure codes: 854 |

CCI: Charlson Comorbidity Index. ECI: Elixhauser Comorbidity Index. Variables based on ICD-10 codes, procedure codes, or medical specialty codes were given the value “1” if one of the specified codes was present, else they were given the value “0”. The string-matching of codes was based on the comparison of the 1:*n* first characters, were *n* is the number of characters in the codes specified in the table (e.g. string-matching for J12 identified all cases with diagnoses J12.0, J12.1, J12.2, J12.3, J.12.8, and J12.9).

[a] Sepsis was identified by clinical and pathogen based sepsis codes. The selection of codes resembles the definition used for sepsis in the largest claims-based quality initiative in Germany – “Initiative Qualitätsmedizin” in 2016 (1).

[b] Categories of the CCI, which had the same definition as corresponding categories of the ECI were excluded. If categories of CCI and ECI were not exactly identical but showed strong overlap, the category that had the stronger univariate relation to hospital mortality in the risk-model was used.

## 2 References

1. Mansky T, Nimptsch U, Cools A, Hellerhoff F. G-IQI | German Inpatient Quality Indicators Version 5.0 - Band 2: Definitionshandbuch für das Datenjahr 2016. Berlin: Technische Universität Berlin; 2016.
