# Supplementary material for: The German Quality Network Sepsis: Evaluation of a Quality Collaborative on Decreasing Sepsis-Related Mortality in a Controlled Interrupted Time Series Analysis
Source: Front Med (Lausanne). 2022 Apr 27;9:882340. doi: 10.3389/fmed.2022.882340 (PMC9094049; doi:10.3389/fmed.2022.882340)
Supplement: Supplementary file 2 [file Data_Sheet_2.pdf]

Supplementary Material 2: Calculation of risk-adjusted mortality

Risk-adjusted mortality was calculated as the risk-standardized mortality rate (RSMR) using established methods (1, 2).

1 Calculation of predicted probability of death

a) Using the risk-adjustment model developed based on the national German diagnosis-related-groups statistics (3) a risk-score RS_{ij} is calculated for each patient j within hospital i is calculated by summing up risk-factors X_k with their respective weights β_k

$$RS_{ij} = \beta_0 + \beta_1 X_1 + \beta_2 X_2 + \cdots + \beta_k X_k$$

b) By transformation of RS_{ij} the individual predicted probability p_{ij} of death is calculated

$$p_{ij} = \frac{\exp(RS_{ij})}{1 + \exp(RS_{ij})}$$

2 Calculation of expected mortality $\boldsymbol{E_i}$ and observed mortality $\boldsymbol{O_i}$ for each hospital $\boldsymbol{i}$

$$E_i = \frac{1}{n_i} \sum_{j=1}^{n_i} p_{ij}$$

$$O_i = \frac{1}{n_i} \sum_{j=1}^{n_i} Y_{ij}$$

Y_{ij} status of patient at discharge (0 = survived, 1 = deceased). n_i number of cases with severe sepsis (or respective subgroup) within hospital i.

3 Calculation of the standardized mortality ratio SMR_i and the risk-standardized mortality rate $RSMR_i$

$$SMR_i = \frac{O_i}{E_i}$$

$$RSMR_i = a * SMR_i$$

a: Mortality of patients with sepsis or the respective subgroup in the German national diagnosis-related-groups statistic of 2015.

4 Calculation of the noise variance $\hat{\sigma}_i^2$ of the $RSMR_i$ and its 95% confidence limit

$$\hat{\sigma}_i^2 = \left(\frac{a}{n_i E_i}\right)^2 \sum_{j=1}^{n_i} p_{ij}(1 - p_{ij})$$

$$CI_{RSMR_i} = RSMR_i \pm 1{,}96\sqrt{\hat{\sigma}_i^2}$$

5 Literature

1. Agency for Health Care Research and Quality Improvement. Quality indicator emprical methods - revised November 2014 [PDF]. 2015 [Available from: http://www.qualityindicators.ahrq.gov/Downloads/Resources/Publications/2015/Empirical_Methods_2015.pdf.
2. DeLong ER, Peterson ED, DeLong DM, Muhlbaier LH, Hackett S, Mark DB. Comparing risk-adjustment methods for provider profiling. Stat Med. 1997;16(23):2645-64.
3. Schwarzkopf D, Fleischmann-Struzek C, Rüddel H, Reinhart K, Thomas-Rüddel DO. A risk-model for hospital mortality among patients with severe sepsis or septic shock based on German national administrative claims data. PLOS ONE. 2018;13(3):e0194371.
