# Supplementary material for: The German Quality Network Sepsis: Evaluation of a Quality Collaborative on Decreasing Sepsis-Related Mortality in a Controlled Interrupted Time Series Analysis
Source: Front Med (Lausanne). 2022 Apr 27;9:882340. doi: 10.3389/fmed.2022.882340 (PMC9094049; doi:10.3389/fmed.2022.882340)
Supplement: Supplementary file 3 [file Data_Sheet_3.pdf]

Supplementary Material 3: Supplementary Tables

Supplementary Table 1. Characteristics of participating hospitals

**Characteristic**	**Participating hospitals, N = 74**
Level of care: Primary care	12 (16.2%)
Secondary care	27 (36.5%)
Tertiary care	18 (24.3%)
University	17 (23%)
Number of inpatient beds	607.5 [400.25, 1138.75]
Duration of intervention (months)	26 [20.75, 26]

Descriptive statistics provided as median [1st quartile, 3rd quartile], or N (%).
